# miR-200b/200a/429 Cluster Stimulates Ovarian Cancer Development by Targeting ING5

**DOI:** 10.1155/2020/3404059

**Published:** 2020-04-22

**Authors:** Wei Guan, Huiling Cui, Ping Huang, Wan Joo Chun, Jin-Won Lee, Heasung Kim, Hua Zou

**Affiliations:** ^1^Cancer Center, Daping Hospital, Third Military Medical University, Chongqing 400042, China; ^2^College of Pharmacy, Yanbian University, Yanji 133002, Jilin Province, China; ^3^Department of Pharmacology, Kangwon National University College of Medicine, Chuncheon 24341, Republic of Korea; ^4^Department of Surgery, Chuncheon Sacred Heart Hospital, College of Medicine, Hallym University, Chuncheon 24253, Republic of Korea

## Abstract

Ovarian cancer is the second most common gynaecological malignancy, and microRNAs (miRNAs) play important role in the cancer development. Here, we found that the level of miR-200b/200a/429 was significantly increased in serum and tumor tissues of patients with stage-I ovarian cancer. Consistent with these results, we detected increased expression levels of miR-200b/200a/429 in ovarian cancer cell lines compared with the human nontumorigenic ovarian epithelial cell line T80. The overexpression of miR-200b/200a/429 in T80 cells stimulated proliferation and caused their growth in soft agar and tumor formation in nude mice. Furthermore, we determined that miR-200b/200a/429 targets inhibitor of growth family 5 (ING5) and that the overexpression of ING5 can block miR-200b/200a/429-induced T80 cell transformation and tumorigenesis. Our findings suggest that miR-200b/200a/429 may be a useful biomarker for the early detection of ovarian cancer and that miR-200b/200a/429 significantly contributes to ovarian cancer development through ING5.

## 1. Introduction

Ovarian cancer is one of the most common gynecologic tumors in the world, with estimated 238,700 new cases and 151,900 deaths in 2012 [1]. The 5-year survival rate of early-stage ovarian cancer patients is more than 92%; however, the 5-year survival rate of late-stage ovarian cancer patients is only 29%, suggesting that early diagnosis is crucial for patient survival [2]. Unfortunately, only approximately 20–25% of patients with ovarian cancer are diagnosed at an early stage [3] due to a lack of early diagnostic markers [2]. Additionally, the molecular mechanism of ovarian cancer development is not fully understood.

MicroRNAs (miRNAs) are short, single-stranded noncoding RNAs that inhibit gene expression at the post-transcriptional level by binding to the 3′-untranslated regions (UTRs) of target gene messenger RNAs. Accumulating evidence shows that miRNAs are aberrantly expressed in tumors and closely correlated with tumor initiation and progression. Notably, the dysregulation of even a single miRNA is sufficient to cause tumor development [4] because one miRNA can target many genes, thereby affecting a large cellular signaling network [5]. Dysregulated expression of the miR-200s family (cluster 1: miR-200b/200a/429 and cluster 2: miR-200c/141) has been indicated in several tumors [6–8]. Interestingly, studies have shown that miRNAs of the miR-200s family play different roles in different progression stages by targeting different genes, even in the same cancer [7]. According to Korpal et al., miR-200s inhibit local invasion by targeting zinc finger E-box-binding homeobox 1/2 (ZEB1/2) but promote lung metastatic colonization by targeting Sec23a in breast cancer [7]. According to Brozovic et al., miR-200s differentially regulate sensitivity to paclitaxel and carboplatin in ovarian cancer [9]. These findings suggest that the role of the miR-200s family at different tumor progression stages is necessary to study. The dysregulated expression of miR-200s was detected previously, and its role in different stages of ovarian cancer progression has been studied, including chemoresistance and metastasis [10, 11]. However, its role in ovarian cancer development is still unclear.

Here, we identified the upregulated expression of miR-200b/200a/429 in the sera of patients with stage-I ovarian cancer compared with healthy controls. The upregulated expression of miR-200b/200a/429 was also identified in stage-I ovarian cancer tissues compared with matched normal tissues. The ectopic expression of miR-200b/200a/429 caused tumor formation in nude mice injected with the nontumorigenic human ovarian epithelial cell line T80 by targeting inhibitor of growth family 5 (ING5). Our findings suggest that miR-200b/200a/429 significantly contributes to ovarian cancer development and has potential as a biomarker for detecting early-stage ovarian cancer.

## 2. Materials and Methods

### 2.1. Materials

miR-200a mimics, miR-200b mimics, miR-429 mimics, and primer set for miR-200a, miR-200b, miR-429, and RNU6 were obtained from RiboBio Co., Ltd. (Guangzhou, China). All cell culture media and fetal bovine sera (FBS) were from Biological Industries (Israel). TRIzol reagent and GeneChip Human Transcriptome Array 2 were purchased from Invitrogen (Carlsbad, CA, USA) and Affymetrix (Santa Clara, CA, USA), respectively. The miRNA Expression Reporter Vector was from Life Technologies (Carlsbad, CA, USA), and the Dual-Luciferase Assay System was from Promega (Madison, WI, USA).

### 2.2. Cell Lines and Clinical Samples

The human ovarian cancer cell lines OVCAR3 and A2780 and the human ovarian epithelial cell line T80 were maintained in DMEM supplemented with 10% fetal bovine serum. All cells were incubated at 37°C in a humidified atmosphere of 95% air and 5% CO_2_.

Ovarian cancer tissues, matched adjacent normal tissues, and sera were collected from 10 patients with newly diagnosed early-stage ovarian cancer at Daping Hospital (Table 1). This research was approved by the Research Ethics Board of Daping Hospital.

### 2.3. RNA Analysis

Total RNA was isolated from cells and tissues using the TRIzol reagent according to the manufacturer's protocol. Mature miR-200a, miR-200b, miR-429, and RNU6 (endogenous control) expression levels were measured by qRT-PCR. The relative expression of miRNAs was normalized to RNU6 expression using the 2^−∆Ct^ method. GeneChip Human Transcriptome Array 2 is used to detect genes whose expression levels are affected by miR-200s in ovarian cancer cells.

### 2.4. Luciferase Reporter Assay

The 3′-UTR segments of ING5 that are predicted to interact with miR-200a/200b/429 were amplified by PCR from human genomic DNA and inserted into the Mlu I and Hind III sites of the miRNA Expression Reporter Vector. For the luciferase reporter assay, cells were seeded into 24-well cell culture plates at a concentration of 1 × 10^4^ per well. The next day, the cells were transfected with the indicated reporter plasmids containing firefly luciferase and either the indicated miRNA mimics or control nucleotides. After 48 hours of transfection, luciferase activity was measured using the Dual-Luciferase Assay System according to the manufacturer's protocol. Firefly luciferase activity was normalized to that of Renilla luciferase.

### 2.5. Soft Agar and Proliferation Assays

Soft agar and proliferation assays were performed as described previously [12].

### 2.6. Animal Experiments

To investigate the effect of miR-200b/200a/429 on ovarian tumor formation, 5 × 10^6^ stably expressing miR-200b/200a/429 T80 cells and their vector control cells in 100 *μ*l of phosphate-buffered saline (PBS) were subcutaneously injected into 6-week-old female nude mice (six mice per group). The experiments were conducted for 6 months. To investigate the role of ING5 in miR-200b/200a/429-induced ovarian tumor formation, stably expressing miR-200b/200a/429 T80 cells were transfected with the ING5 expression vector or an empty vector and 5 × 10^6^ cells in 100 *μ*l of PBS were subcutaneously injected into 6-week-old female nude mice (seven mice per group). The experiments were conducted for 6 months.

### 2.7. Statistical Analysis

All data are presented as the mean ± standard deviation, and significant differences between treatment groups were analyzed by Student's *t*-test. Differences were considered statistically significant at a *p* value less than 0.05.

## 3. Results

### 3.1. miR-200b/200a/429 Expression Was Significantly Increased in Early-Stage Ovarian Cancer

**Figure 1:**
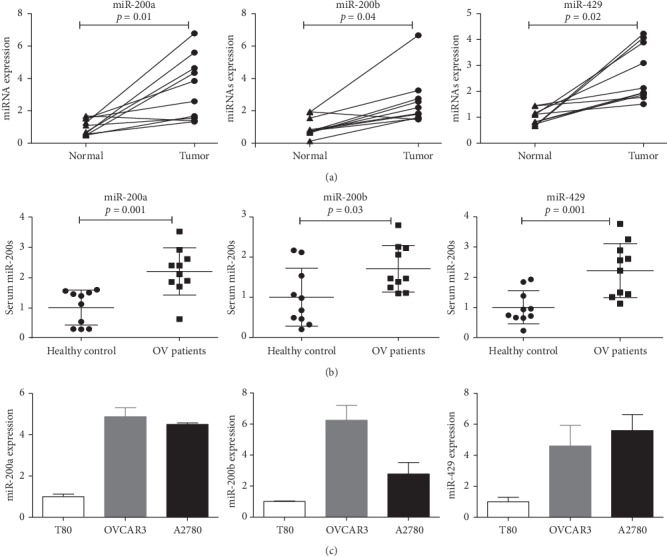
The expression of miR-200b/200a/429 was significantly upregulated in ovarian cancer. (a) The expression of miR-200b/200a/429 significantly increased in stage-I ovarian cancer tissues compared with their matched adjacent normal tissues (*n* = 10). (b) Serum levels of miR-200b/200a/429 higher in patients with stage-I ovarian cancer (*n* = 10) than healthy controls (*n* = 10). (c) The expression of miR-200b/200a/429 significantly increased in ovarian cancer cells compared with T80 ovarian epithelial cells.

To investigate the correlation between miR-200b/200a/429 expression and ovarian cancer development, we compared the expression level of miR-200b/200a/429 between early-stage ovarian cancer tissues and normal ovarian tissues. As shown in Figure 1(a), miR-200b/200a/429 expression was significantly increased in stage-I ovarian cancer tissues compared with matched adjacent normal tissues. In addition, we found that the miR-200b/200a/429 level was significantly higher in the sera of stage-I ovarian cancer patients than in healthy controls (Figure 1(b)). Finally, we determined that the expression of miR-200b/200a/429 was higher in ovarian cancer cell lines than in T80 nontumorigenic ovarian epithelial cells (Figure 1(c)). These findings suggest that the increased expression of miR-200b/200a/429 may be associated with ovarian cancer development.

### 3.2. Overexpression of miR-200b/200a/429 Stimulates Ovarian Tumorigenesis

**Figure 2:**
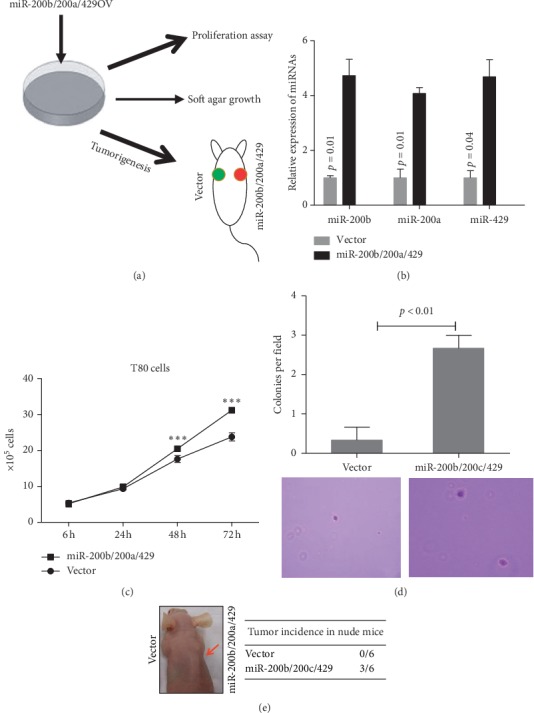
Overexpression of miR-200b/200a/429 stimulated the transformation of T80 ovarian epithelial cells. (a) Schematic diagram of the experiments. (b) The expression of miR-200a, miR-200b, and miR-429 was significantly increased in miR-200b/200a/429 stably transfected T80 cells. (c) Overexpression of miR-200b/200a/429-accelerated T80 cell proliferation. ^*∗∗∗*^*p* < 0.001 compared to the vector control group. (d) Overexpression of miR-200b/200a/429 caused the anchorage-independent growth of T80 ovarian epithelial cells in soft agar. (e) Overexpression of miR-200b/200a/429 caused the tumor formation of T80 cells in nude mice. miR-200s, miR-200b/200a/429; miR-200s in, inhibitor of miR-200b/200a/429.

To investigate whether the increased expression of miR-200b/200a/429 is involved in ovarian cancer development, we overexpressed miR-200b/200a/429 in T80 nontumorigenic human ovarian epithelial cells and then subjected the cells to cell proliferation, anchorage-independent growth, and tumor formation assays (Figure 2(a)). Our results showed that the overexpression of miR-200b/200a/429 (Figure 2(b)) significantly stimulated T80 cell proliferation compared with the vector control groups (Figure 2(c)). In addition, the overexpression of miR-200b/200a/429 caused T80 cells to readily form foci in soft agar, indicating that miR-200b/200a/429 promotes the transformation of ovarian epithelial cells (Figure 2(d)). More importantly, the overexpression of miR-200b/200a/429 led to T80 nontumorigenic ovarian epithelial cells forming tumors in 50% of nude mice after 6 months of cell injection (Figure 2(e)). Taken together, these findings suggest that the increased expression of miR-200b/200a/429 significantly contributes to ovarian cancer development.

### 3.3. ING5 Is a Target of miR-200b/200a/429

**Figure 3:**
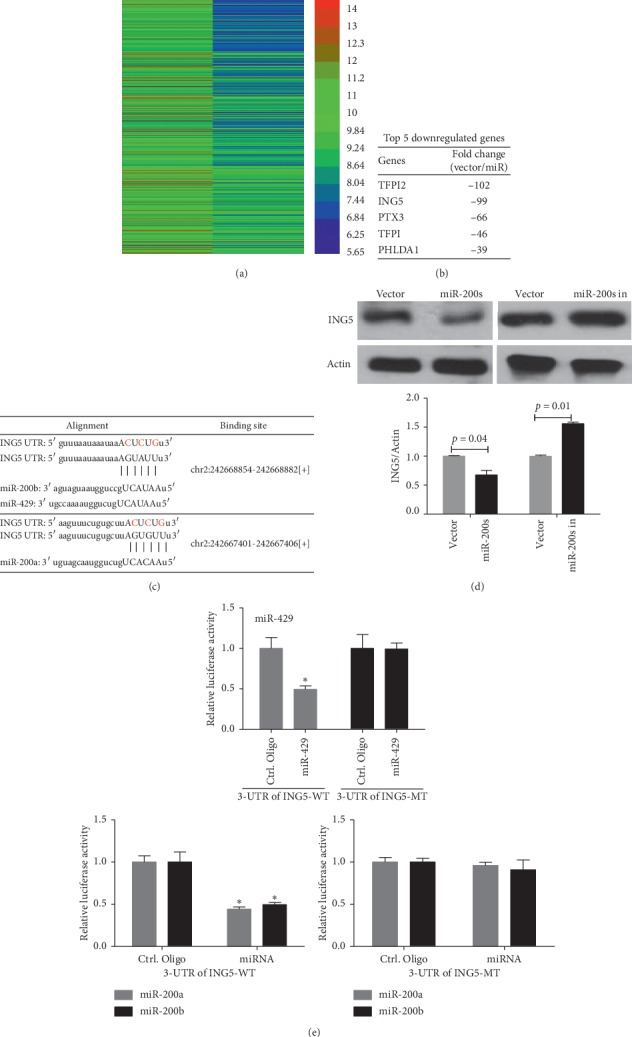
ING5 is a target gene of miR-200b/200a/429 in ovarian epithelial cells. (a) Heatmap showing the downregulated genes in T80 cells by miR-200b/200a/429. mRNA array was performed using miR-200b/200a/429 cluster overexpressing T80 cell and their vector control cells (one replicate). (b) Top 5 genes that were downregulated by miR-200b/200a/429 in T80 cells. (c) Sequence alignment of miR-200b/200a/429 with the 3′-UTR of ING5. (d) miR-200b/200a/429 negatively regulated the expression of ING5. (e) miR-200b/200a/429 inhibits ING5 promoter-regulated gene expression. T80 cells were cotransfected with the 3′-UTR luciferase reporter construct (wild-type or mutant-type) of ING5 and the indicated nucleotides. After 48 hours of transfection, the cells were subjected to a luciferase activity assay.

To investigate the underlying mechanism of miR-200b/200a/429 in ovarian tumorigenesis, we performed a gene array using miR-200b/200a/429-overexpressing T80 cells and their control cells. As shown in Figure 3(a), we observed many genes that were downregulated by miR-200b/200a/429 in T80 cells (Figure 3(a)). Then, we used miRNA target prediction algorithms (starbase.sysu.edu.cn) to determine whether the 3′-UTR of the top 5 downregulated genes (Figure 3(b)) has a sequence that can bind to miR-200b/200a/429. As shown in Figure 3(c), we determined that the 3′-UTR of ING5 contains sites that can bind to miR-200a, miR-200b, and miR-429. Next, we investigated whether miR-200b/200a/429 is directly involved in the inhibition of ING5 protein expression. Our data showed that the overexpression of miR-200b/200a/429 significantly inhibited ING5 protein expression levels in T80 cells compared with the vector control, whereas the inhibition of miR-200b/200a/429 increased ING5 protein expression levels in T80 cells compared with the vector control (Figure 3(d)). Furthermore, to determine whether the expression regulation of ING5 by miR-200b/miR-200a/429 is dependent on binding to the ING5 3′-UTR region, a three-nucleotide mutation was inserted into the ING5 3′-UTR (red), as indicated in Figure 3(c). Our data showed that the overexpression of miR-200b, miR-200a, or miR-429 significantly repressed luciferase activity that is regulated by ING5 3′-UTR (Figure 3(e)). However, the 3′-UTR mutation of ING5 completely abrogated the effect of overexpressing miR-200b, miR-200a, or miR-429 on luciferase activity in 293T cells (Figure 3(e)). Cumulatively, these data suggest that miR-200b/200a/429 suppress the expression of ING5 in the ovary by directly targeting their 3′-UTR.

### 3.4. miR-200b/200a/429 Stimulates Ovarian Tumorigenesis through ING5

**Figure 4:**
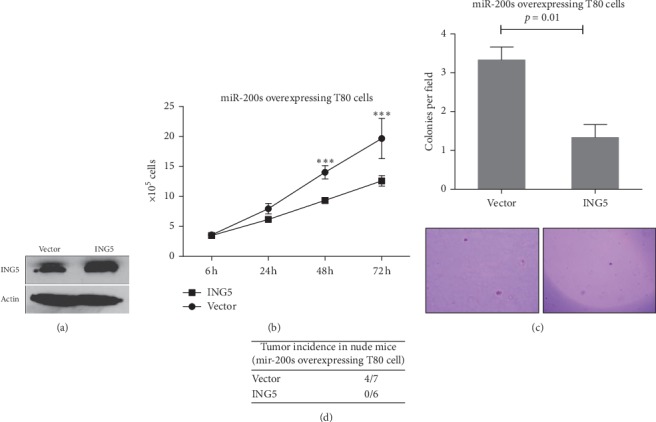
miR-200b/200a/429 plays an oncogenic role through ING5. (a) ING5 protein level was dramatically increased by transfection of the ING5 expression plasmid. Stably expressing miR-200b/200a/429 T80 cells were transfected with the ING5 expression plasmid or an empty vector. After 72 hours of transfection, cells were subjected to western blot analysis. (b) Overexpression of ING5 significantly inhibited miR-200b/200a/429-overexpressing T80 cell proliferation. Stably expressing miR-200b/200a/429 T80 cells were transfected with the ING5 expression plasmid or an empty vector and then subjected to a cell proliferation assay. ^*∗∗∗*^*p* < 0.001 compared to the vector control group. (c) Overexpression of ING5 significantly inhibited the foci formation of miR-200b/200a/429-overexpressing T80 cells in soft agar. (d) The miR-200b/200a/429-induced tumor formation of T80 cells in nude mice was blocked by ING5 overexpression.

To investigate whether ING5 is involved in miR-200b/200a/429-induced ovarian tumorigenesis, we overexpressed ING5 in miR-200b/200a/429-stably overexpressing T80 cells (Figure 4(a)) and then performed cell proliferation, soft agar, and *in vivo* tumor formation assays. Our data showed that the overexpression of ING5 (Figure 4(a)) significantly inhibited miR-200b/200a/429-overexpressing T80 cell proliferation (Figure 4(b)) and foci formation in soft agar (Figure 4(c)). Importantly, the overexpression of ING5 completely blocked the tumor formation of miR-200b/200a/429-overexpressing T80 cells in nude mice (Figure 4(d)). These findings suggest that miR-200b/200a/429 plays its oncogenic role through ING5 in ovarian cancer.

## 4. Discussion

In the present study, we demonstrated the role of miR-200b/200a/429 in ovarian cancer development. Our data showed that miR-200b/200a/429 was significantly increased in early-stage ovarian cancer tissues and in the sera of early-stage ovarian cancer patients compared with normal ovarian tissues and healthy controls, respectively. In addition, the overexpression of miR-200b/200a/429 caused the transformation and tumor formation of T80 nontumorigenic ovarian epithelial cells. Similar to our results, Mateescu et al. reported that the overexpression of miR-200b/200a/429 can markedly enhance transformation and tumor formation in K-Ras-transformed fibroblasts [8]. These findings clearly indicate that miR-200b/200a/429 plays role in ovarian cancer development as an oncogenic miRNA and may have potential as a biomarker for the diagnosis of early-stage ovarian cancer. However, further verification is needed in a large sample size of early-stage ovarian cancer patients.

We also clarified the oncogenic mechanism of miR-200b/200a/429 in ovarian cancer development. ING5 is a member of the ING family and is a tumor suppressor gene [13]. According to Zheng et al., the downregulation of ING5 is closely correlated with ovarian carcinogenesis, ovarian cancer metastasis, and angiogenesis [14]. In this study, for the first time, we found that ING5 expression was negatively regulated by miR-200b/200a/429 in the ovary. Our data showed that the overexpression of miR-200b/200a/429 in ovarian epithelial cells led to the suppression of ING5 expression; conversely, the inhibition of miR-200b/200a/429 further upregulated ING5 expression. Additionally, luciferase reporter gene experiments showed that miR-200b/200a/429 directly targets the 3′-UTR of ING5. In addition, our data showed that the overexpression of ING5 in T80 cells dramatically abrogated the miR-200b/200a/429 overexpression-induced stimulation of cell proliferation, anchorage-independent growth in soft agar, and tumor formation in nude mice. These findings clearly suggest that miR-200b/200a/429 mediates tumorigenesis and cell proliferation by inhibiting ING5 in ovarian cancer. However, the mechanism of ING5 in ovarian cancer development is not clear and needs further study.

In summary, our findings suggest that miR-200b/200a/429 is an oncogenic miRNA that significantly contributes to ovarian cancer development by targeting ING5. Additionally, miR-200b/200a/429 may be a useful candidate biomarker for the detection of early-stage ovarian cancer.

## Figures and Tables

**Table 1:** Characteristics of patients with HGSOC.

Patient number	Age	FIGO stage	Lymph node metastasis
1	63	І	No
2	55	І	No
3	72	І	No
4	38	І	No
5	56	І	No
6	40	І	No
7	47	І	No
8	36	І	No
9	52	І	No
10	41	І	No

HGSOC, high-grade serous ovarian carcinoma.

## Data Availability

The data used to support the findings of this study are available from the corresponding author upon request.
